# Atrial cardiomyopathy: From healthy atria to atrial failure. A clinical consensus statement of the Heart Failure Association of the ESC

**DOI:** 10.1002/ejhf.3782

**Published:** 2025-08-05

**Authors:** Jerremy Weerts, Otilia Țica, Julia Aranyo, Christian Basile, Angelina Borizanova‐Petkova, Josip Andjelo Borovac, Massimiliano Camilli, Martin Eichenlaub, Emiliano Fiori, Tim Van Loon, Coenraad Withaar, Diana Zakarkaitė, Matthias D. Zink, Marianna Adamo, Alberto Aimo, Elena Arbelo, Felipe Bisbal Van Bylen, Dimitrios T. Farmakis, Dobromir Dobrev, Jelena Čelutkienė, Michael Böhm, Andrew Coats, Marco Metra, Giuseppe Rosano, Frank Ruschitzka, Antoni Bayes‐Genis, Dipak Kotecha

**Affiliations:** ^1^ Department of Cardiology Cardiovascular Research Institute Maastricht, Maastricht, University Medical Centre Maastricht The Netherlands; ^2^ Heart Institute, Germans Trias i Pujol University Hospital Badalona Spain; ^3^ Department of Cardiovascular Sciences University of Birmingham Birmingham UK; ^4^ Emergency County Clinical Hospital of Bihor Oradea Romania; ^5^ Department of Advanced Biomedical Sciences University of Naples ‘Federico II’ Naples Italy; ^6^ Division of Cardiology, Department of Medicine Karolinska Institutet Stockholm Sweden; ^7^ Department of Emergency Medicine Medical University Sofia Sofia Bulgaria; ^8^ Cardiology Clinic, UMHAT ‘Tsaritsa Yoanna‐ISUL’ Sofia Bulgaria; ^9^ Cardiovascular Diseases Department University Hospital of Split (KBC Split) Split Croatia; ^10^ Department of Cardiovascular Medicine Fondazione Policlinico Universitario A. Gemelli IRCCS Rome Italy; ^11^ Department of Cardiovascular and Pulmonary Sciences Catholic University of the Sacred Heart Rome Italy; ^12^ Department of Cardiology and Angiology Medical Center, University of Freiburg Bad Krozingen Germany; ^13^ Department of Clinical and Molecular Medicine Sapienza University of Rome Rome Italy; ^14^ Department of Biomedical Engineering Cardiovascular Research Institute Maastricht, Maastricht University Maastricht The Netherlands; ^15^ Department of Cardiology, University of Groningen University Medical Center Groningen Groningen The Netherlands; ^16^ Clinic of Cardiac and Vascular Diseases, Faculty of Medicine, Vilnius University Vilnius Lithuania; ^17^ Ortenau‐Klinikum Lahr, Academic Teaching Hospital University of Freiburg Lahr Germany; ^18^ Cardiology Unit, ASST Spedali Civili di Brescia, Department of Medical and Surgical Specialties, Radiological Sciences, and Public Health University of Brescia Brescia Italy; ^19^ Cardiology Division, Fondazione Toscana Gabriele Monasterio, Health Sciences Interdisciplinary Center, Scuola Superiore Sant'Anna Pisa Italy; ^20^ Arrhythmia Section, Cardiology Department, Hospital Clínic, Universitat de Barcelona Barcelona Spain; ^21^ Institut d'Investigació August Pi i Sunyer Barcelona Spain; ^22^ Centro de Investigación Biomédica en Red de Enfermedades Cardiovasculares Madrid Spain; ^23^ Heart Failure Unit, Department of Cardiology Athens University Hospital Attikon, National and Kapodistrian University of Athens Medical School Athens Greece; ^24^ Institute of Pharmacology, Faculty of Medicine, University Duisburg‐Essen Essen Germany; ^25^ Department of Integrative Physiology Baylor College of Medicine Houston TX USA; ^26^ Department of Medicine Montreal Heart Institute and Université de Montréal Montréal QC Canada; ^27^ Centre of Innovative Medicine Vilnius Lithuania; ^28^ Universitätsklinikum des Saarlandes, Klinik für Innere Medizin III, Saarland University Homburg Germany; ^29^ Kardiologie, Angiologie und Internistische Intensivmedizin Homburg Germany; ^30^ Heart Research Institute Sydney NSW Australia; ^31^ CAG Cardiovascular, St George's University Hospital London UK; ^32^ Department of Human Sciences and Promotion of Quality of Life San Raffaele University of Rome Rome Italy; ^33^ IRCCS San Raffaele Roma Italy; ^34^ Center for Translational and Experimental Cardiology, Department of Cardiology, University Hospital Zurich, University of Zurich Zurich Switzerland; ^35^ Julius Center University Medical Center Utrecht The Netherlands; ^36^ Birmingham NIHR Biomedical Research Centre, University Hospitals Birmingham NHS Foundation Trust Birmingham UK

**Keywords:** Atrial cardiomyopathy, Atrial fibrosis, Heart failure, Atrial fibrillation, Imaging, Electrocardiography

## Abstract

The importance of atrial cardiomyopathy (AtCM) as a specific clinical entity is increasingly recognized. Past definitions have varied, and the lack of consistent cut‐offs for imaging parameters and biomarkers have limited clinical utility to diagnose and track AtCM progression. While research has mainly focused on AtCM in the context of atrial fibrillation, emerging evidence underscores its relevance in remodelling and development of heart failure. The aim of this consensus document was to provide a contemporary framework for AtCM, evolve the definitions of AtCM and atrial failure for more widespread clinical use, and help to direct emerging research and future clinical trials. Supporting the work of early career researchers, this consensus document evaluates diagnostic markers and summarizes the underpinning mechanisms, clinical characteristics and prognostic impact of AtCM. Our objective was to bring together new translational scientific progress, catalyse future research and enable clinical application to facilitate better management, for example in patient groups where aggressive control of risk factors or comorbidities could prevent AtCM progression. We redefined AtCM as a graded disorder that includes electrical dysfunction of the atria along with evidence of either mechanical atrial dysfunction, atrial enlargement and/or atrial fibrosis. Atrial failure is the end‐stage manifestation of AtCM, characterized by progressive structural, electrophysiological and functional changes. Earlier identification, risk stratification and ongoing research into therapeutic options have the potential to prevent the clinical consequences of AtCM and atrial failure, including adverse patient outcomes and poor quality of life associated with atrial fibrillation and heart failure.

## Introduction

The atria play a significant role in overall cardiac function, as well as systemic and pulmonary haemodynamics.^1^ Beyond their impact on ventricular filling, they serve as volumetric reservoirs supporting effective ventricular contraction, and house pacemaker cells and other crucial components of the cardiac conduction system. Moreover, they secrete natriuretic peptides, which are pivotal in regulating fluid homeostasis and vascular tone. Notably, the atrial myocardium is susceptible to various cardiac and non‐cardiac diseases and is particularly sensitive to loading conditions and extra‐cardiac factors.

There is a growing realization of the importance of atrial disease, of which the most evident consequences are heart failure (HF), a syndrome where cardiac function is insufficient for the individual's needs,^2^, ^3^ and atrial fibrillation (AF), characterized by uncoordinated electrical activation that impedes atrial contraction.^4^ AF is the most common sustained arrhythmia,^5^ and increases the risk of stroke and other thromboembolic outcomes, HF and mortality.^6^, ^7^ The lack of direct correlation in many studies between episodes of AF and subsequent thromboembolism is indicative that AF can be an intermittent manifestation of underlying cardiomyopathy.^8^, ^9^ For example, in patients with continuous pacemaker monitoring, subclinical AF >6 min is detected in only 15% of patients during the month before an embolic event.^10^ Rather than waiting for incident AF or HF to occur, earlier identification of atrial remodelling may provide an opportunity to intercede. Left atrial (LA) reservoir strain (LASr) is more closely associated with stroke and dementia risk than AF,^11^, ^12^ and in patients with HF and preserved ejection fraction (HFpEF), mechanical atrial dysfunction is an independent and better predictor than AF to stratify for adverse clinical outcomes.^13^ There may also be treatment implications; for example, in patients with an embolic stroke of undetermined source, rivaroxaban in the NAVIGATE‐ESUS trial had no overall advantage over aspirin in reducing the recurrence of stroke or systemic embolism,^14^ except in patients with an LA diameter exceeding 46 mm.^15^

The term atrial cardiomyopathy (AtCM) was initially proposed in 1972 to describe patients with first‐degree heart block and ectopic supraventricular rhythms progressing to persistent atrial standstill.^16^ Subsequent definitions and consensus documents have moved the field forward scientifically, initially based on correlated histological changes to provide a solid framework for AtCM diagnosis.^17^, ^18^ Whilst there are genetic variants proposed in the pathology of AtCM in rare patients,^19^ genetic predisposition in the majority of those with AtCM is complex and multifactorial, with polygenic risk markers associated with both structural and functional LA parameters.^20^, ^21^ The ‘common soil hypothesis’^22^ suggests that stressors such as ageing, cardio‐metabolic risk factors and concomitant diseases promote (sub)clinical atrial disease through mechanisms such as inflammation, endothelial and microvascular dysfunction, fibrosis, hypercoagulability, and atrial stretch. Underlying atrial disease therefore becomes a specific clinical entity and a potentially targetable indicator of adverse prognosis, even in the absence of clinically‐detected AF or HF.

The primary aim of the present consensus document was to propose a definition of AtCM that could be applied with ease in clinical practice, and be a scaffold for emerging research that could inform the development of this field (*Graphical Abstract*). This document evolves prior definitions, reflects the current state‐of‐the‐art in clinical research, and was designed to be applied not just by electrophysiology or HF specialists, but earlier and more widely across different medical specialties. The approach was developed following a number of in‐person and virtual meetings coordinated by the Committee on Atrial Disorders from the Heart Failure Association (HFA) of the European Society of Cardiology (ESC). This included early career investigators involved in basic, translational or clinical research on the mechanisms or clinical manifestations of AtCM. These early career researchers performed and presented reviews of the available literature within each section to inform the consensus approach. Our objective was to stimulate this new field within cardiology, underpinning research and clinical trials that can improve patient care and prevent the development of AF, HF and other adverse outcomes.

## Proposed definition of atrial cardiomyopathy and atrial failure

In light of the presently available body of evidence, we propose defining AtCM as ‘*electrical and mechanical dysfunction of the atria, resulting from underlying pathological changes that lead to atrial enlargement or atrial fibrosis, with the potential to produce clinical consequences*’.

**Figure 1:**
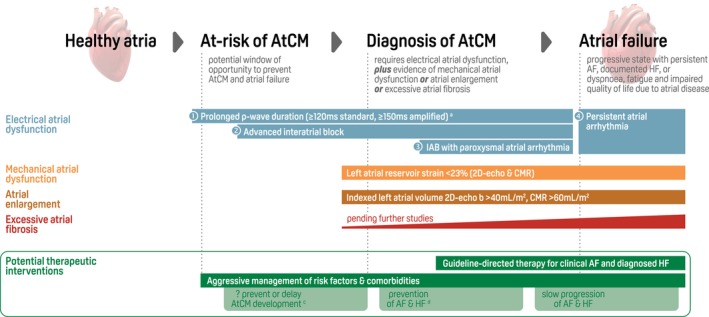
Consensus approach to diagnosis of atrial cardiomyopathy (AtCM) and prevention of atrial failure. AtCM is defined as electrical and mechanical dysfunction of the atria, resulting from underlying pathological changes that lead to atrial enlargement or atrial fibrosis, with the potential to produce clinical consequences. The diagnostic cut‐points for AtCM in this document will need to be updated with new studies; although somewhat arbitrary, they represent the consensus group's best approximation of a workable AtCM diagnosis at this time. 2D, two‐dimensional; AF, atrial fibrillation; CMR, cardiac magnetic resonance; CVD, cardiovascular disease; ESC, European Society of Cardiology; HF, heart failure; IAB, inter‐atrial block. ^a^Numbers in circles represent the AtCM P‐wave score; see *Table* *2* for other relevant P‐wave abnormalities. ^b^For women aged >65 years, the 2D‐echo AtCM threshold for indexed left atrial volume is >48 ml/m^2^. ^c^Clinical trials are needed specifically in patients at risk or with AtCM. ^d^The 2024 ESC guidelines on AF have class I recommendations on primary prevention of AF for maintaining optimal blood pressure, normal weight and an active lifestyle, and avoidance of binge drinking.^4^ The 2021/2023 ESC guidelines on HF have class I recommendations on primary prevention of HF for treatment of hypertension; statins in those at high risk of CVD; sodium–glucose co‐transporter 2 inhibitors in patients with diabetes and CVD, or diabetes and chronic kidney disease; finerenone in patients with diabetes and chronic kidney disease; and counselling against sedentary habit, obesity, smoking and alcohol abuse.^2^, ^3^

Consensus was built on the need for both clinical simplicity and also to ensure specificity for AtCM. Hence the diagnosis of AtCM requires demonstration of *electrical atrial dysfunction* in combination with evidence of either *mechanical atrial dysfunction*, *atrial enlargement* and/or *excessive atrial fibrosis* (*Figure* *1*). Combining markers in this way may also avoid over‐diagnosis, particularly as a variety of diseases can cause similar features of atrial disruption. The possible manifestations of electrical and mechanical atrial dysfunction are discussed below. Further, we comment on the cut‐offs for atrial enlargement, emerging methods to detect atrial fibrosis, biomarkers that could in future help to refine risk and diagnosis of AtCM, and the molecular underpinning of AtCM that could reveal future targets for therapeutic intervention.

Atrial cardiomyopathy can be due to primary atrial disease without other relevant initial cardiac abnormalities, or secondary to existing ventricular and/or valvular disease. Similar to ventricular myopathy, AtCM has a spectrum of severity and the combination of different atrial disease markers will likely indicate a higher risk of progression. AtCM can manifest through abnormalities in atrial geometry, wall structure and function, which are typically detected by cardiac imaging techniques. However, the severity and consistency of these abnormalities lack universally accepted, and prognostically documented cut‐offs. Furthermore, reference values are only available for a limited number of atrial markers.^23^, ^24^ Underpinning this iterative proposed AtCM framework is the ability for surrogate biomarkers to allow for earlier and more accurate detection of AtCM. Most of the atrial markers studied have shown association with risk of stroke and thromboembolism even in the absence of AF, including LA size^25^ and advanced inter‐atrial block (IAB).^23^, ^26^ However, there is clear need for more research to identify the most reproducible and easily applicable markers for routine clinical practice.

More pragmatic and hence earlier diagnosis of AtCM has the potential to prevent atrial failure, the end‐stage of AtCM manifestation occurring due to progressive structural, electrophysiological and functional changes.^27^ Atrial failure is characterized by clinical manifestations such as persistent AF or documented HF, with dyspnoea, fatigue and impaired quality of life attributable (at least in part) to atrial disease. A combination of the proposed AtCM markers presumably leads to a higher risk of atrial failure. This presents an opportunity for timely risk stratification and management in patients with AtCM, or those at risk of AtCM, to prevent or delay atrial failure. Pending future clinical trials in AtCM, at present this could include aggressive management of underlying risk factors and comorbidities.

### Future directions

Clinical validation studies of the proposed AtCM definition framework will be required, along with iterative updates over time to reflect new research findings, new technology, and changing patient substrate and comorbidity patterns. There is limited evidence currently on the role of natriuretic peptides and other biomarkers to stratify risk of AtCM and atrial failure. Well‐powered studies are essential to develop approaches that have diagnostic accuracy for AtCM and can be easily implemented in routine practice. It is likely that additional manifestations of atrial failure remain undiscovered and warrant further exploration. Robust studies are needed that can inform on strategies to prevent progression of AtCM to atrial failure.

## Diagnostic criteria for electrical atrial dysfunction

**Figure 2:**
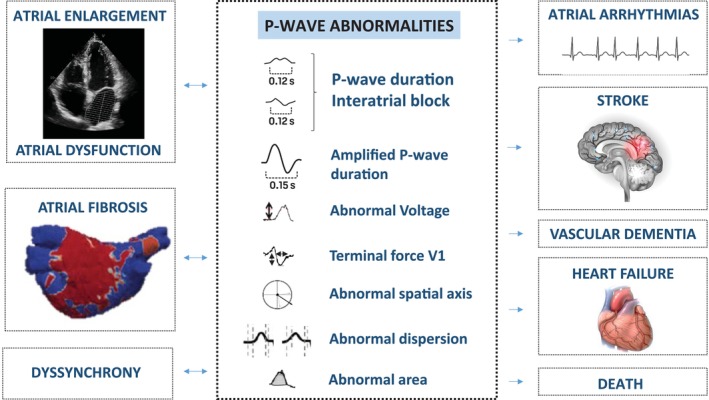
Electrocardiographic markers indicating atrial disorders. Visual summary of P‐wave abnormalities measured on an electrocardiogram. Most P‐wave abnormalities have been associated with imaging modalities of atrial cardiomyopathy, as well as cardiovascular adverse events.

Surface electrocardiography (ECG) is a widely available technique to assess cardiac electrophysiology in humans. The ECG has been the hallmark of AF diagnosis.^28^ The ECG can also be used to predict future incidence of AF or HF,^29^, ^30^ with ECGs displaying distinct features once atrial dysfunction develops (*Figure* *2*). The presence of paroxysmal AF is an opportunity to intercede in the progression of AtCM at a ‘pre‐atrial failure’ stage, but many of these ECG markers occur even earlier, allowing for pre‐emptive action to address the underlying drivers of AtCM.

### P‐wave abnormalities in atrial cardiomyopathy

Under normal conditions, the sinoatrial impulse propagates from the right to the left atrium predominantly through the Bachmann bundle, allowing fast and synchronic biauricular depolarization.^31^ A normal P‐wave has duration <110 ms, is positive in inferior leads, with a biphasic morphology only in V1. Additionally, the normal P‐wave usually has a voltage >0.1 mV and an axis between 0 and 75°. Any alteration in electrophysiological properties, atrial structure or atrial function are typically reflected by changes in P‐wave morphology, highlighting the importance of P‐wave analysis to indicate AtCM.

Several P‐wave abnormalities have been reported.^31^, ^32^ One of the most studied parameters is the presence of IAB, caused by a conduction delay or Bachmann's bundle block.^33^ In this context, the depolarization of the left atrium occurs caudo‐cranially via septal and coronary sinus fibers, causing significant electromechanical asynchrony. IAB is defined as a P‐wave duration ≥120 ms and is classified into three degrees: partial (P‐wave duration >120 ms with positive polarity and usually bimodal morphology in the inferior leads); advanced (P‐wave duration ≥120 ms with Bachmann's bundle block showing biphasic configuration in ≥2 inferior leads due to caudo‐cranial depolarization); and intermittent (when a variable degree of block appears).^34^ The diagnosis of IAB is frequently associated with LA remodelling and fibrosis. IAB, especially advanced IAB, has been associated with an increased risk for adverse outcomes, such as AF, ischaemic stroke, cognitive impairment, HF and mortality.^23^, ^35^, ^36^, ^37^, ^38^ The association between IAB and new‐onset supraventricular arrhythmias, including AF, is known as Bayes syndrome.^39^ The risk of AF has been reported to increase twofold and fourfold in patients with partial and advanced IAB, respectively.^40^

Although the majority of evidence comes from advanced IAB, other P‐wave indexes have also been linked with AtCM. Furthermore, IAB is typically associated with an advanced AtCM stage and may be absent in patients with a diffuse or patchy disease substrate.^41^ Therefore, it is important to combine IAB assessment with other sensitive ECG parameters to evaluate earlier stages of AtCM. Measurement of the P‐wave duration allows the quantification of interatrial conduction slowing in patients with AtCM and is also associated with ischaemic stroke, cognitive impairment, HF and mortality.^42^, ^43^ However, the reported cut‐off values for various clinical outcomes are highly variable. In a large cohort study, both a very short and a very long P‐wave duration were associated with the occurrence of death, stroke and AF.^44^ This might be explained by patients with advanced AtCM having hidden or underestimated P‐wave prolongation, as low‐amplitude P‐wave components may not be visualized on the 12‐lead ECG using standard settings.^41^, ^43^ Amplification of the 12‐lead ECG might be useful to appreciate low‐amplitude components, allowing for measurement of the amplified P‐wave duration that is highly correlated with LA hypertension, mechanical dysfunction, and invasively‐measured low‐voltage areas.^45^ Other techniques that enhance ECG analysis and aid AtCM detection include additional leads and higher sample rates for advanced post‐processing analysis.^46^, ^47^, ^48^, ^49^, ^50^, ^51^

Additional P‐wave indexes, such as voltage, axis, morphology, dispersion, area, and terminal force in V1, have also been described as risk factors for AF, stroke, HF, dementia and mortality.^52^, ^53^, ^54^ Each ECG parameter has advantages and disadvantages in determining AtCM (*Table* *1*). Besides these ‘classical’ ECG parameters, artificial intelligence has the potential to significantly improve AtCM diagnosis, as demonstrated for new‐onset AF, HF, and mortality.^55^

**Table 1:** Strengths and weaknesses of P‐wave parameters to predict atrial cardiomyopathy

P‐wave parameter	Strength	Weakness
IAB	Evidence **+++** for advanced IAB in advanced AtCM stages Easy to measure	Less evidence for partial IAB Moderate sensitivity
P‐wave duration	Evidence ++ for early AtCM stages Easy to measure	Undersensing of low‐amplitude P‐wave components using standard settings Signal‐to‐noise ratio No established pathological cut‐off
P‐wave voltage	Evidence Easy to measure	Undersensing of low‐amplitude P‐wave components using standard settings Signal‐to‐noise ratio
P‐wave axis	Evidence **++** Easy to measure	Cannot be calculated in the presence of advanced IAB
P‐wave terminal force V1	Evidence **++** Only one lead is needed	Difficult to measure
P‐wave dispersion	Evidence	Long recording ECG Difficult to measure
P‐wave area	Easy to measure	Little evidence Difficult to measure
P‐wave morphology in orthogonal leads	Evidence	Difficult to measure

AtCM, atrial cardiomyopathy; ECG, electrocardiography; IAB, inter‐atrial block.

### P‐wave characteristics to diagnose atrial cardiomyopathy

**Figure 3:**
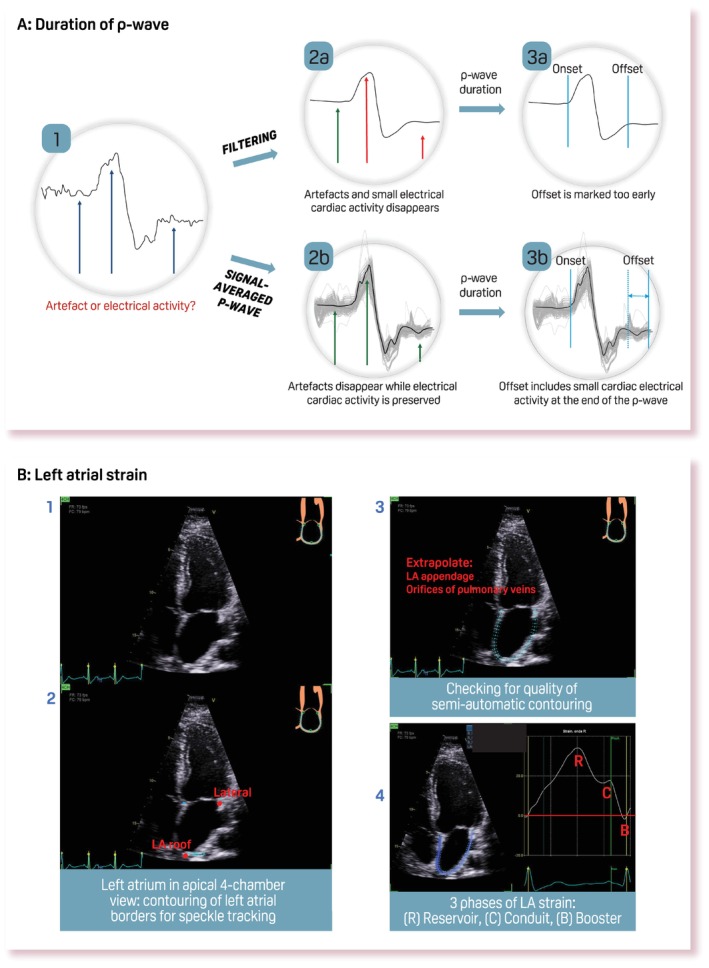
Electrocardiogram (ECG) and imaging evaluation for atrial cardiomyopathy. (*A*) Steps for calculating P‐wave duration (see *Appendix* *1* for details). Step 1: an unfiltered P‐wave in V1 shows small changes in electrical activity (arrows) which can be a result of small artefacts (electrical noise, muscle activity, or others). Step 2a: by applying routine filters for a standard ECG recording, the macro‐shape of the P‐wave becomes visible and many artefacts will disappear, as well as small changes in atrial electrical activity. Step 3a: parts of the P‐wave with small atrial electrical activity and low amplitudes may be neglected in marking the earliest and last atrial electrical activity. Step 2b: a signal‐averaged P‐wave consists of a long recording of unfiltered ECG (light grey lines). By calculating the average P‐wave signal (black line) of these unfiltered ECGs, randomly distributed artefacts disappear while small changes in atrial electrical activity are preserved. Step 3b: this allows exact marking of the earliest and last atrial electrical activity. (*B*) Steps for calculating left atrial (LA) strain (see *Appendix* *2* for details). LA reservoir strain: the reservoir phase of LA strain represents the positive wave with a peak during systole and negative deflection in the phase of passive LA filling. The obtained value is always positive. LA conduit strain: the second positive peak following the P‐wave in the pre‐contraction phase. In sinus rhythm, this phase occurs from the time of mitral valve opening through diastasis, until the onset of LA contraction. In subjects with atrial fibrillation, the LA conduit phase has the same value as the LA reservoir strain but it is negative. LA booster strain: this occurs from the onset of LA contraction until end‐diastole in patients with sinus rhythm. It always has a negative value.

P‐wave abnormalities may identify a subset of patients with previously undetected AtCM, allowing for risk stratification and the potential for specific preventive or therapeutic measures. We propose a score based on the P‐wave, where the parameters with the most evidence provide the most weight (*Table* *2*). Explanation on how to perform P‐wave measurements is elaborated in *Appendix* *1* and *Figure* *3*. Intermediate risk (score of 1 or 2) is represented by patients with prolonged P‐wave duration or IAB, but without clinical atrial arrhythmias. The management of these earlier stages of AtCM (preceding clinical AF) remains an area of relative knowledge gap,^56^, ^57^, ^58^ although it may be possible to prevent or delay AtCM progression and inhibit progression to atrial failure. The use of anticoagulants and rhythm control strategies in this group remains controversial and requires further investigation. It is conceivable that diligent comorbidity and risk factor management of patients with intermediate risk may slow down atrial remodelling and subsequently prevent the onset of AF and/or HF.^59^ A score of ≥3 is obtained in the presence of paroxysmal or persistent AF, highlighting the need for anticoagulation or antiarrhythmic strategies to effectively manage the condition. In the presence of persistent AF, the 12‐lead ECG becomes less useful, and prognostic stratification tools become more relevant. These include, for example, continuous monitoring to determine AF burden or ventricular response.^60^ Patients with AF but without demonstrable AtCM are of special interest to deepen the understanding of the AtCM framework, including AF due to specific triggers such as critical illness, alcohol intake and genetic background.

**Table 2:** Atrial cardiomyopathy P‐wave score

P‐wave disturbances to identify electrical atrial dysfunction and AtCM risk	Score
Absence of any changes in P‐wave parameters	0
Prolonged P‐wave duration (≥120 ms on standard ECG or ≥150 ms on amplified 12‐lead ECG)^a^	1
Advanced inter‐atrial block (P‐wave ≥120 ms plus biphasic morphology in ≥2 inferior leads) without clinical atrial arrhythmia	2
Advanced inter‐atrial block with episodes of paroxysmal atrial arrhythmia	3
Persistent atrial arrhythmia	4

The diagnosis of AtCM according to this consensus approach requires documented electrical atrial dysfunction (AtCM P‐wave score of 1 or more) in combination with evidence of either mechanical atrial dysfunction, atrial enlargement, or excessive atrial fibrosis. Patients at risk of AtCM (score of 1 or 2) deserve proactive management of comorbidity and risk factors to prevent or revert progression of structural and mechanical dysfunction. A total score of 3 or more warrants an evaluation of thromboembolic risk, and the use of anticoagulation or antiarrhythmic strategies as per clinical practice guidelines.

AtCM, atrial cardiomyopathy; ECG, electrocardiography.

^a^ The presence of partial inter‐atrial block (P‐wave ≥120 ms with positive polarity and usually bimodal morphology in inferior leads), abnormal P‐wave voltage (≤0.1 mV), axis (<0° or >75°), P‐wave terminal force in V1 (>40 mm/ms or >4 mV/ms) and dispersion (>40 ms) also constitute an AtCM P‐wave score of 1.

### Future directions

Improved ability to distinguish anatomical versus functional factors underpinning specific P‐wave disorders would help to redefine ECG‐based risk prediction of AtCM. The appropriate ablation strategy in patients with AF^61^ and advanced atrial failure, and its ability to modify the substrate and progression of the disease, requires further investigation. In addition, clarification is needed on the role of invasive techniques, such as atrial high‐density electro‐anatomical mapping, to diagnose AtCM and for prognostic stratification. Artificial intelligence algorithms are being developed to automate P‐wave analysis,^62^ and could play an increasing role in future clinical practice once reliability, accuracy and diagnostic/prognostic prediction are clarified.

## Diagnostic criteria for mechanical atrial dysfunction

**Figure 4:**
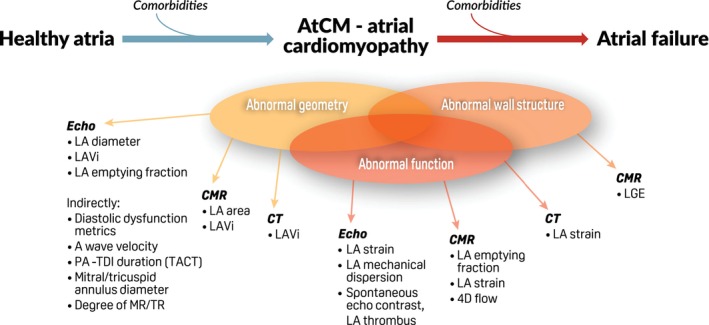
Evolution of atrial markers obtained by multimodal imaging. Multimodality imaging represents a useful approach to intercept subclinical changes in the left atrium, together with the surface electrocardiogram and serum biomarkers. Due to limited data currently, only indexed left atrial (LA) volume and LA reservoir strain are incorporated in the present atrial cardiomyopathy (AtCM) diagnostic framework. 4D, four‐dimensional; CMR, cardiac magnetic resonance; CT, computed tomography; LAVi, left atrial volume index; LGE, late gadolinium enhancement; MR, mitral regurgitation; PA‐TDI (TACT), P‐wave on surface electrocardiogram to peak A'‐wave on tissue Doppler imaging (also known as the total atrial conduction time); TR, tricuspid regurgitation.

Atrial remodelling occurs in parallel with electrical dysfunction and leads to persistent changes in atrial size and function, often associated with increased susceptibility to atrial arrhythmias.^63^ In contrast to the left ventricle, the left atrium presents a pressure–volume loop pattern with three main functional components: filling (or reservoir), passive emptying (or conduit), and active emptying (or booster pump). Each of these components is progressively hampered in diastolic dysfunction, with a gradual reduction of LA functional reserve. Atrial remodelling can be distinguished into a functional component (consisting of an impaired function without necessarily an increment in size) and structural component (with altered geometry and/or wall fibrosis). Imaging techniques, most notably echocardiography and cardiac magnetic resonance (CMR), as well as cardiac computed tomography (CCT) are able to capture different aspects of atrial remodelling (*Figure* *4*).^17^, ^27^, ^64^

Left atrial function, particularly atrial contraction, is reflected by the amplitude and width of transmitral atrial‐induced flow velocity (a wave).^65^ The ratio with peak early mitral velocity (E/a ratio) has classically been used as an index of LA and left ventricular (LV) pressures. More recently, LA strain analysis has been introduced to estimate reservoir, conduit and booster function,^66^, ^67^ for which practical recommendations have been released by scientific societies^66^ (‘how‐to’ explanations in *Appendix* *2* and *Figure* *3*). LA strain depends on the preload, afterload, and intrinsic atrial contractility. Specifically, LASr is tightly coupled to LV longitudinal shortening and is strongly influenced by LV strain, LV end‐diastolic pressure and pulmonary capillary wedge pressure.^68^, ^69^, ^70^, ^71^ LA strain is useful for the assessment LV filling pressures, defining the severity of diastolic dysfunction, and establishing the diagnosis of HFpEF.^72^ Its role has been demonstrated for risk stratification in several disease settings.^13^, ^73^ For instance, LASr was shown to be a determinant for morbidity (e.g. HF) and cardiovascular mortality in the general population,^74^ as well as acute HF.^71^ Evaluation of LA function may help identify patients at high risk of atrial arrhythmias^75^ and risk of ischaemic stroke,^76^, ^77^ similar to use in those undergoing AF catheter ablation to establish propensity to maintain sinus rhythm.^78^ Given the relatively easy assessment of LASr and the available evidence as prognostic factor compared to other LA strain assessments, only LASr is included in this proposed definition of AtCM.

Normal ranges for two‐dimensional (2D) echocardiography‐derived LA strain have been established.^79^, ^80^, ^81^ Thresholds for abnormal LASr are proposed at <23%, with LA booster strain threshold <5% for ages 40–65 years and <8% in those >65 years.^68^, ^69^, ^70^, ^71^, ^80^ Three‐dimensional (3D) strain analysis can also be conducted, however normative values remain unclear and the impact of different pathologies warrants further validation.^82^ Other functional metrics of potential value are LA emptying fraction,^83^ LA mechanical dispersion index^84^ and LA stiffness index,^85^ whereas tissue Doppler‐based strain assessment has been superseded by speckle‐tracking approaches.^86^ CMR‐based feature tracking analysis provides information on atrial deformation comparable to echocardiographic speckle tracking. The proposed CMR‐derived cut‐offs for abnormal LA function are <23% for LASr and <8% for LA booster strain.^87^, ^88^, ^89^, ^90^ *Table* *3* provides common atrial imaging values to indicate AtCM,^79^, ^80^, ^88^, ^89^, ^90^, ^91^, ^92^, ^93^, ^94^, ^95^, ^96^, ^97^, ^98^, ^99^, ^100^, ^101^ and multimodality atrial imaging has recently been reviewed in an ESC clinical consensus statement.^102^

**Table 3:** Atrial imaging parameters used in clinical practice to define structural and functional alterations

	Echocardiography	Cardiac magnetic resonance imaging	Cardiac computed tomography
Strengths	Quick and easy to conduct with wide access Timely assessment of LA dimensions, LV/RV function, atrio‐ventricular valves 3D analysis Speckle‐tracking analysis	High spatial and contrast resolution Better reproducibility and lower variability Identification of LA appendage thrombus, LV/RV function, atrio‐ventricular valves 3D analysis Possibility of feature‐tracking analysis Tissue characterization (LGE, ECV, T mapping); Useful in pre‐procedural planning (catheter ablation)	High spatial resolution Identification of LA appendage thrombus Presence of atrial septal defect or patent foramen ovale; atrial wall thickness and dimension assessment Better reproducibility and lower variability 3D analysis Possibility of feature‐tracking analysis Tissue characterization (late iodine enhancement, ECV), epicardial adipose tissue characterization Useful in pre‐procedural planning (catheter ablation, LA appendage occlusion, tricuspid/mitral valve intervention)
Limitations	High interobserver variability (lower for 3D analysis) Limited spatial resolution Limited by acoustic windows Tissue characterization not available	Limited availability Use of contrast medium (gadolinium)	Limited availability Use of iodinated contrast medium Exposure to radiation Data on normal values

Proposed thresholds of abnormality suggestive for atrial cardiomyopathy			
**Left atrium**			
LA diameter (anteroposterior)	>40 mm in men >38 mm in women^91^	>42 mm in men >41 mm in women^92^	>45 mm in men >44 mm in women^93^
LA maximum volume indexed by BSA	2D: >40 ml/m^2^ >48 ml/m^2^ in women >65 years 3D: >41 ml/m^2^ >48 ml/m^2^ in patients >65 years^80^	>60 ml/m^2^ ^94^	2D: >73 ml/m^2^ 3D: >78 ml/m^2^ ^95^
LA area	>22 cm^2^ for SR patients >28 cm^2^ for AF patients^96^	>28 cm^2^ ^92^, ^97^	
LA reservoir strain	2D: <23%^79^, ^80^, ^98^	<23%^88^, ^89^, ^90^	No studies in sufficiently large populations to propose optimal cut‐offs
LA conduit strain	2D: <12% <9% in patients >65 years^80^	<21%^99^
LA booster strain	2D: <8%^80^	<8%^88^, ^89^, ^90^
LA emptying fraction	2D: <48% 3D: <43%	<46%^80^, ^94^
**Right atrium**			
RA volume indexed by BSA	>36 ml/m^2^ in men^100^ >30 ml/m^2^ in women^100^	>61 ml/m^2^ in men >54 ml/m^2^ in women^94^	>89 ml/m^2^ ^95^
RA area	>22 cm^2^ ^101^ in men >18 cm^2^ ^101^ in women	>27 cm^2^ in men >24 cm^2^ in women^97^	>22 cm^2^ ^100^

Note that studies vary in the methodology of assessment, inclusion or exclusion of appendage or venous structures, and automated or post‐processing approaches.

3D, three‐dimensional; BSA, body surface area; ECV, extra‐cellular volume; LA, left atrial; LGE, late gadolinium enhancement; LV, left ventricular; RA, right atrial; RV, right ventricular.

## Diagnostic criteria for left atrial enlargement

Left atrial enlargement and geometrical changes typically result from chronically elevated LA pressures in non‐AF patients.^65^, ^103^ 2D LA volume indexed for body surface area (LAVi) represents the most extensively investigated parameter of LA remodelling, providing important prognostic implications,^104^ particularly in patients with HF or AF.^105^, ^106^ In those with HF, adverse LA remodelling is associated with higher risk of death and HF hospitalization, with increases in LA diameter preventable through up‐titration of angiotensin‐converting enzyme inhibitors or angiotensin receptor blockers,^107^ and sodium–glucose co‐transporter 2 inhibitors.^108^ LA size also predicts AF, stroke and mortality in the general population.^109^, ^110^ The proposed cut‐off for diagnosing LA enlargement based on 2D echocardiography is LAVi >40 ml/m^2^, with a higher threshold of >48 ml/m^2^ for women aged >65 years^80^ (*Table* *3*). 3D echocardiography and CMR have better spatial resolution and can enhance the accuracy of volume estimates. The proposed cut‐offs for diagnosing atrial enlargement using 3D echocardiography LAVi are >41 ml/m^2^ (>48 ml/m^2^ for women >65 years),^80^ and >60 ml/m^2^ using CMR‐derived LAVi.^94^ Atrial enlargement has typically been measured at maximal volume on echocardiography, however minimal LA volume may be more highly associated with clinical outcomes in patients with HF,^111^, ^112^ albeit with greater measurement variability.^113^

## Diagnostic criteria for atrial fibrosis

Atrial fibrosis is a primary manifestation of AtCM that is linked to the electrical, mechanical and structural alterations discussed, and associated with development of atrial failure, AF, HF and adverse events. Scientific understanding of atrial fibrosis and its relevance is advancing.^114^ Notably, atrial fibrosis manifests in various forms, including reactive and replacement fibrosis.^115^ CMR can detect atrial fibrosis through late gadolinium enhancement (LGE),^116^ but this is limited by the spatial resolution of current CMR scanning in relation to the thin atrial wall. Furthermore, a variety of different acquisition and post‐processing protocols are available which differ in both the amount and regional distribution of detected LA fibrosis.^117^, ^118^ Small studies suggest that LA LGE ≥10–15% can predict incident atrial arrhythmias, stroke and arrhythmia recurrence after pulmonary vein isolation,^119^, ^120^, ^121^ but further research is clearly warranted prior to widespread clinical use. LASr correlates with the extent of histological atrial fibrosis in advanced HF patients, highlighting the possibility of echocardiography as a surrogate measure.^122^, ^123^ Due to limited histological validation,^124^ it remains unclear whether detected atrial fibrosis, which relies on threshold settings of LGE wash‐out, primarily identifies larger areas of replacement fibrosis or is also sensitive to reactive (endomysial and perimysial) fibrosis. CMR and CCT can be valuable tools for planning AF ablation^125^ and quantifying epicardial adipose tissue, which plays an important role in atrial remodelilng.^126^

### Future directions

Further study is needed on the relationship between LA structural and functional alterations and AF/stroke occurrence, hard endpoints (cardiovascular mortality, HF hospitalization), as well as the response to drug therapy in HF with reduced ejection fraction (HFrEF) and HFpEF, and to percutaneous interventions for aortic and mitral valve disease. There is a clear requirement to identify cut‐offs for LA size and function that have prognostic value or can stratify risk of AtCM. This includes optimal clinical thresholds that indicate atrial remodelling, for example with combined electrical/structural markers such as the total atrial conduction time that measures duration from depolarization to active LA contraction.^127^ Histological validation of imaging modalities to quantify atrial fibrosis will be important to further our mechanistic understanding of AtCM, and to quantify any reversal of atrial fibrosis in response to medical therapy and other interventions. This includes sodium–glucose co‐transporter 2 inhibitors and glucagon‐like peptide‐1 receptor agonists which have known antifibrotic properties, but where the impact on atrial fibrosis development and progression is unclear.^128^

## Role of the right heart and comorbidities in atrial cardiomyopathy

A notable gap exists in our understanding of the interactions between the right heart and AtCM. Similarly, the mechanisms whereby systemic conditions and non‐cardiac comorbidities may influence cardiac remodelling and affect the electrophysiological and mechanical properties of the atria have not been fully elucidated.

The interplay between right heart abnormalities and LA dysfunction has been explored in patients with HFpEF.^109^ The term ‘disproportionate LA myopathy’ was introduced to describe a condition where LA dysfunction was more pronounced than expected based on observed LV dysfunction. Right heart dysfunction through enhanced left‐to‐right atrial interaction and heightened pericardial constraint may be a contributor to LA myopathy.^109^ The exacerbation and progression of cardiovascular conditions with adverse cardiac remodelling because of systemic, non‐cardiac comorbidities is well established, and is particularly evident in HFpEF and AF.^129^, ^130^, ^131^, ^132^, ^133^, ^134^ Physiological ageing, coupled with common comorbidities such as hypertension, obesity, diabetes mellitus and valvular disease can all contribute to atrial remodelling.^17^, ^135^ The obesity phenotype of HFpEF is characterized by right ventricular dilatation and LV diastolic dysfunction through pericardial constraint and ventricular interdependence.^136^ Tricuspid regurgitation, causing right ventricular volume overload, may also lead to HFpEF and atrial myopathy.^137^, ^138^ Comorbidities have been linked to reduced LA and right atrial strain parameters in paroxysmal AF, particularly when more than three conditions are present, including hypertension, diabetes, coronary artery disease, obesity, age >65 years, moderate‐to‐severe mitral regurgitation and kidney disease.^139^

Whether incidence or progression of AtCM can be prevented through effective management of AtCM precursors requires dedicated clinical trials. In the interim, it may be reasonable to encourage early aggressive management of risk factors and comorbidities in selected patients to prevent or reduce the progression of AtCM. This is similar to the AF‐CARE approach detailed in the 2024 ESC guidelines on AF. In patients with clinical AF, there were sufficient trials for class I recommendations to reduce arrhythmia recurrence and prevent AF progression by better management of hypertension, diabetes mellitus and obesity, in addition to enhancing exercise capacity and restriction of alcohol intake.^4^

### Future directions

Collectively, systemic conditions and non‐cardiac comorbidities have the potential to significantly influence cardiac remodelling and can impact the electrophysiological and mechanical properties of the atria. However, the mechanisms by which these effects occur are not fully understood and warrant further exploration through mechanistic studies, and potentially preclinical or experimental investigations.

## Future molecular targets for atrial cardiomyopathy

New mechanisms can shed light on AtCM and the bidirectional relationship between AF and HF and its therapeutic targets.^140^, ^141^ Here we provide promising insights into the evolving research on the development, diagnosis or progression of AtCM.

### Biomarkers

B‐type natriuretic peptides are used in clinical practice to diagnose HF,^142^ although their utility for AtCM is unclear. The Atrial Cardiopathy and Antithrombotic Drugs in Prevention after Cryptogenic Stroke (ARCADIA) trial used B‐type natriuretic peptide (>250 pg/ml) or P‐wave terminal force to indicate AtCM, but was stopped early due to futility for the comparison between apixaban versus aspirin to prevent recurrent stroke.^143^ LA wall stretch also prompts atrial natriuretic peptide (ANP) release. The left atrium in a failing heart exhibits abundant yet defective ANP synthesis,^144^, ^145^ leading to a relative deficit of the active ANP form.^146^ Altered processing and clearance of natriuretic peptides increase susceptibility to volume overload, acute decompensation^147^ and progression of HF.^148^ ANP exerts anti‐hypertrophic effects on the left ventricle^149^ and may stimulate autophagy and mitophagy,^150^, ^151^ and it exhibits anti‐arrhythmic properties, reducing the likelihood of re‐entry and preventing AF maintenance.^152^ In AF, ANP synthesis is initially stimulated but reverses with arrhythmia progression, with a negative correlation between ANP production and atrial collagen deposition. In contrast, excessive ANP production can lead to amyloid fibril deposition, isolated atrial amyloidosis and AtCM.^153^, ^154^ Mid‐regional proANP has emerged as a potentially‐specific biomarker for AF^155^ and AtCM in HFpEF,^156^, ^157^ although distinct thresholds require further research.

Bone morphogenetic protein 10 (BMP10) belongs to the transforming growth factor‐beta family and is a novel biomarker for atrial remodelling and AF. As an atrial‐specific ligand, higher plasma levels may reflect more severely altered atrial structure.^158^, ^159^ Elevated BMP10 levels have been linked to increased risk of adverse cardiovascular events,^160^, ^161^, ^162^, ^163^, ^164^ higher AF recurrence after rhythm control, and late postoperative AF after cardiovascular surgery.^165^ The precise interaction between BMP10 and AF or HF remains unclear, but may not be directly causal,^166^ hence the need for further investigation in its role as a risk stratifier for AtCM.^160^, ^161^, ^163^, ^167^

### Atrial fibrosis

The pathophysiological mechanisms leading to atrial fibrosis are diverse, involving stretch‐induced activation of fibroblasts, systemic inflammatory processes, activation of coagulation factors, and fibro‐fatty infiltrations.^168^ While activated fibroblasts are the principal cells involved in collagen synthesis, other cell types such as atrial myocytes, adipocytes, and immune cells also play crucial roles in sensing environmental factors that contribute to atrial fibrosis.^169^, ^170^ On a microscopic scale, diffuse endomysial fibrosis causes discontinuous conduction, longitudinal dissociation, and endo‐epicardial dissociation of electrical activity during AF.^171^, ^172^ Larger, patchy fibrosis areas on a macroscopic scale are more likely to anchor macro re‐entrant circuits. Further relevant histopathological findings have previously been summarized.^18^ The heterogeneity of atrial fibrosis mechanism complicates the development of effective therapeutic strategies, but also presents an opportunity for innovation.^128^

### Inflammasome

Metabolic and cardiovascular disorders, such as hypertension, obesity, diabetes, gut dysbiosis, chronic kidney disease and sepsis, are known to stimulate the activation of nucleotide‐binding oligomerization domain‐like receptor protein 3 (NLRP3).^173^, ^174^, ^175^, ^176^, ^177^, ^178^, ^179^, ^180^ Increased NLRP3 inflammasome activity in atrial cardiomyocytes, immune cells^181^ and fibroblasts^182^ mediates active interleukin‐1β and interleukin‐18 release and the promotion of a fibro‐inflammatory cycle. This can contribute to atrial and ventricular structural and electrical remodelling, promoting AtCM and an AF‐maintaining substrate by affecting ion channel functionality and Ca^2+^ handling. Mechanistic studies are needed to elucidate the causal relationship between inflammasome activation in atrial cells and AtCM, as well as the efficacy of NLRP3‐specific inhibitors.^177^, ^179^, ^183^, ^184^, ^185^, ^186^, ^187^, ^188^ It should be noted that clinical trials of conventional anti‐inflammatory drugs such as colchicine have demonstrated a mixed response,^189^ with the largest randomized trials showing no significant prevention of AF.^190^, ^191^

### Electrical and anatomical remodelling

Cardiomyopathies associated with arrhythmia and diastolic dysfunction often share intrinsic cardiac triggers that may underly AtCM.^192^ Electrical remodelling, usually defined as shortening of the atrial effective refractory period and action potential duration, contributes to atrial arrhythmogenesis by promoting re‐entrant activity.^193^ Novel therapeutical targets have been identified, including inhibition of phosphodiesterase (PDE) type‐8, particularly the atrial‐selective PDE8B2 isoform,^194^ and upregulation of the Ca^2+^‐dependent SK current.^195^ Reactive oxygen species‐dependent activation of Ca^2+^/calmodulin‐dependent protein kinase IIδc could also be an important target to prevent adverse remodelling.^196^ In HF, elevated filling pressures impose mechanical stress and chronic overload on the atria. This leads to varying degrees of atrial remodelling, but the precise molecular mechanisms remain elusive.^197^ Genetic and non‐genetic alterations in active and passive force generation at the sarcomere level can contribute to electrical dysfunction via altered myofilament Ca^2+^ sensitivity, sarcomere integrity, impaired contractility and disturbed coordination of signalling molecules, all of which could induce and maintain AF by causing re‐entry‐promoting atrial remodelling.^198^, ^199^, ^200^ Atrial cardiomyocytes exhibit Ca^2+^ handling and structural, but not electrical, remodelling in HFrEF patients,^201^ unless AF co‐occurs.^201^ Ca^2+^‐handling abnormalities that cause cellular triggered activity are likely the major trigger of AF in HFrEF,^201^ with AF subsequently producing electrical remodelling that promotes its maintenance.^202^ Mainly in HFrEF, reduced phosphorylation of cardiac myosin‐binding protein C leads to contractile dysfunction and Ca^2+^‐cycling abnormalities, contributing to cardiac arrhythmias.^203^, ^204^ In HFpEF, increased myocardial stiffness, titin isoform alterations and fibrosis result in heterogeneous conduction that, when present in the atria, are a substrate for the persistence and propagation of atrial arrhythmias.^205^, ^206^, ^207^ Defective mitochondrial oxidative capacity, particularly fatty acid metabolism, appears to be present within atrial cardiomyocytes in AF^208^ and HFpEF.^209^ Restoring the fatty acid metabolism oxidation presents an opportunity to tackle both disorders,^209^, ^210^ although glucose metabolism and comorbidities such as obesity are confounders. Further understanding these electro‐mechanical mechanisms will be crucial to delineate the common and distinct triggers of these conditions, and their role in the pathogenesis of AtCM.

### Epicardial adipose tissue

Epicardial adipose tissue has been implicated in the pathogenesis of AF and HFpEF,^211^, ^212^ and could influence progression of AtCM^211^, ^213^, ^214^ through atrial fat infiltration, release of pro‐inflammatory and pro‐fibrotic mediators, oxidative stress, altered ion currents, gap junction modulation, and autonomic dysfunction.^211^, ^215^, ^216^ Patients with HFpEF have more epicardial fat than controls or those with HFrEF,^214^, ^217^ and more atrial epicardial adipose tissue when AF is co‐occurring.^212^ The browning of these adipocytes may offer protection against AF and other cardiovascular conditions by mitigating inflammation and improving metabolic profiles,^218^ contrasting with that observed in HFpEF.^219^ The negative cardiometabolic effects of adipocytes may be more pronounced in women than men, potentially increasing the risk of AtCM and a HFpEF phenotype in women.^220^

## Clinical application, gaps in evidence and future directions

To diagnose and stratify AtCM, we suggest a comprehensive assessment of clinical status, evaluation of electrical atrial dysfunction using ECG to assess for P‐wave abnormalities, and imaging to determine mechanical atrial dysfunction and atrial enlargement. The latter is most commonly interrogated with echocardiography (including speckle‐tracking analysis), with CMR considered as a complementary examination, especially when better resolution or tissue characterization is clinically indicated. It is essential to integrate the assessment of the left atrium with thorough evaluation of LV systolic and diastolic function, valve disease and the right heart. This comprehensive approach will allow the exclusion of other structural heart alterations as a cause of symptoms, and put the potential diagnosis of AtCM in context. The presence of any one AtCM marker (electrical atrial dysfunction, mechanical atrial dysfunction, atrial enlargement or excessive atrial fibrosis) should prompt this diagnostic work‐up. However, electrical atrial dysfunction plus one of the structural markers is required under this proposed definition to confirm AtCM, in order to avoid over‐diagnosis. This is a key development over previous AtCM definitions, with the enhanced specificity designed to stimulate new clinical research and trials that can demonstrate opportunities to prevent AtCM or its progression.

Regardless of whether a patient is deemed at risk or with AtCM, they should undergo detailed screening for HF using imaging and natriuretic peptides, and appropriate ECG monitoring for atrial arrhythmias as outlined in the respective ESC guidelines.^2^, ^3^, ^4^ The role that gender plays on risk stratification or management of AtCM is unclear, although studies have demonstrated that women have a higher prevalence and more extensive regions of low‐voltage zones in the left atrium than men^221^, ^222^ that could indicate more advanced underlying cardiomyopathy by the time of diagnosis or treatment. AtCM with confirmation of either HF or persistent AF provides a diagnosis of atrial failure that requires therapy for each condition as per clinical guidelines,^2^, ^3^, ^4^ with the potential for prognostic benefit. Considering the high risk of adverse outcomes in patients with AtCM and atrial failure, monitoring by a cardiologist with either HF or AF expertise is advisable, depending on local infrastructure. Important evidence gaps and possible future research directions for the AtCM diagnostic framework are summarized in *Table* *4*,^101^, ^223^, ^224^, ^225^, ^226^, ^227^, ^228^, ^229^, ^230^, ^231^, ^232^, ^233^, ^234^ with the aim of galvanizing AtCM research, and providing clearer diagnostic cut‐offs for clinical use and enhancing their implementation in routine practice.

**Table 4:** Important evidence gaps in atrial cardiomyopathy and suggested future directions

Evidence gaps	Future directions
Expected clinical course of the AtCM spectrum and relevant clinical markers	More population and mechanistic studies are needed to identify the natural course of AtCM and its relation to clinically relevant outcomes. Specifically, are there features or markers that indicate a higher risk of stroke, thromboembolism or progressive HF development in individuals with or at risk of AtCM? How do common comorbidities (such as hypertension, obesity, diabetes mellitus and coronary artery disease) or lifestyle (smoking, obesity and exercise) as well as treatment impact the course of AtCM? Do women have the same trajectory for AtCM development and progression as men after accounting for biology and diagnostic/treatment biases? Which underlying causes lead to AtCM independent of left ventricular, right heart or valvular dysfunction? Secondary functional mitral or tricuspid valve regurgitation due to atrial remodelling (enlargement) may indicate progressed atrial failure, but what are important modifiable factors in this process?^101^, ^223^, ^224^
AtCM diagnostic framework is not clinically validated	Well‐powered studies are essential to improve this diagnostic framework for AtCM. Evaluation and refinement of the AtCM diagnostic framework in retrospective and prospective studies requires careful balancing for age, gender, cardiovascular risk factors and history of cardiac disease. Registry data on HF are essential to comprehensively assess AtCM/atrial failure prevalence across the ejection fraction spectrum. Attention is needed on the interaction and ability to distinguish between AtCM‐related atrial failure and HFpEF phenotypes.
Serum biomarkers to facilitate AtCM diagnosis	Studies exploring the role of a range of natriuretic peptides, corin, and BMP10 biomarkers in diagnosing AtCM or identifying targets for therapy, including relevant clinical cut‐points.
Easily obtained or clinically reliable parameters to facilitate AtCM diagnosis	Explore the clinical relevance of implementing ambulatory rhythm devices, such as wearables, photoplethysmography signal processing, and implantable devices, within the AtCM framework.^225^, ^226^, ^227^, ^228^, ^229^ The role of invasive techniques such as atrial high‐density electro‐anatomical mapping for the diagnosis of AtCM and prognostic stratification requires further investigation. Which artificial intelligence algorithms can improve the AtCM diagnostic framework and selection for future therapies?
Reliability of imaging parameters when scanned in the presence of AF	Reproducibility of imaging parameters is poor in the context of AF, with further data awaited on alternative approaches such as the index‐beat method of acquisition following two preceding R‐R intervals of similar duration.^230^
Uncover additional manifestations of atrial failure	Further studies on the relationship between left atrial structural and functional alterations and AF/stroke occurrence, hard endpoints (mortality, hospitalization), as well as response to optimized medical therapy.
The role of right heart dysfunction in AtCM	Right heart dysfunction and its impact on AtCM development requires further dedicated imaging studies. How does the interaction between the left and right atria impact AtCM? A range of imaging modalities could improve insights into AtCM and the right atrium.
Patient selection for future clinical trials and the threshold of AtCM markers to initiate therapy and prevent disease progression	Identification of AtCM markers with strong prognostic value that can aid patient selection for clinical trials to improve outcomes. Randomized controlled trials assessing the use of anticoagulants in individuals with AtCM but without AF are needed to enhance our understanding of the impact of AtCM on stroke and thromboembolism.
Functional properties underlying different stages within the AtCM framework	Studies on relevant atrial fibrosis cut‐off values by cardiac magnetic resonance imaging to diagnose AtCM are needed. The digital twin approach^231^ could enhance (pre‐)clinical research by providing a platform for studying the isolated and interconnected effects of the specific disease subsets that are relevant to the AtCM spectrum. This involves validating and translating these models for practical clinical applications, necessitating further research to seamlessly integrate theoretical frameworks into real‐world healthcare practices. Examples include improved ability to distinguish anatomical versus functional factors underpinning specific P‐wave disorders to redefine ECG‐based risk prediction for AtCM, or to better phenotype AtCM on tissue property levels such as atrial myocardial compliance and contractility.
The effect of therapies on AtCM substrates and clinical outcomes	The potential benefit of using anticoagulant and antiarrhythmic therapy in patients with pathological P‐wave parameters in the absence of atrial arrhythmias is controversial and requires further investigation. What kind of clinical benefit can be achieved with anti‐remodelling treatments (such as neprilysin or sodium–glucose co‐transporter 2 inhibitors) in patients with early stages of AtCM? The appropriate ablation strategy in patients with AF and advanced AtCM, and its ability to modify substrate and disease progression, requires specific investigation. What patient outcomes are relevant for those with AtCM, what could predict atrial failure, and what is the optimal way to monitor and subsequently intervene in selected populations?
Targeting of specific underlying mechanisms to halt or reverse AtCM progression and prevent atrial failure	Future research should focus on elucidating the inflammasome effectors on cardiac electrophysiology and AF pathogenesis, and delineating their role in cardiomyocyte inflammatory signalling with fibroblasts and immune cells. Are selective inhibitors for these pathways effective at preventing development of AF or HF? Studies are needed to assess the efficacy of inhibitors in the electrical remodelling pathway, explore the regulation of atrial ion channels and calcium handling, and identify genetic predispositions to HF (for example, through PITX2^232^, ^233^, ^234^). Future research should investigate the influence of epicardial adipose tissue on atrial fibrosis, sex‐dependent mechanisms and the relationship with inflammation. Patients with AF and without AtCM who eventually develop atrial failure are of special interest to deepen the understanding of the AtCM framework.

AF, atrial fibrillation; AtCM, atrial cardiomyopathy; BMP10, bone morphogenetic protein 10; ECG, electrocardiography; HF, heart failure; HFpEF, heart failure with preserved ejection fraction.

## Conclusions

Atrial cardiomyopathy is a progressive condition that starts from apparently healthy atria, traverses a subclinical phase of atrial disease, and continues to an end‐stage of atrial failure that has irreversible consequences for the patient. This paper, coordinated by the HFA of the ESC and supporting early career investigators, was designed to stimulate the field, coalesce research targets and facilitate future clinical trials to prevent progression of AtCM. We propose a new pragmatic framework for AtCM based on electrophysiology, imaging, biomarkers, and clinical status. Straightforward criteria are suggested to confirm electrical atrial dysfunction, mechanical atrial dysfunction and atrial enlargement in the clinical environment. Atrial fibrosis is an important part of the proposed AtCM definition, with further preclinical and clinical evidence required to understand its role in AtCM stratification. Earlier detection of AtCM utilizing a range of developing and promising targets could harness novel therapeutics, or repurposing of existing therapy, to prevent overt atrial failure, AF, HF and their sequalae.

## Appendix 1

**How to perform P‐wave analysis**

The P‐wave duration marks the beginning and end of atrial electrical activity. Hence, exact marking is of utmost importance for any further analysis of P‐wave parameters, such as inter‐atrial block (IAB) and others. The measurement of P‐wave duration can be performed using various techniques.

Routinely, P‐wave duration is measured via a standard 12‐lead electrocardiogram (ECG) (e.g. recorded 6–12 s, 200 Hz sample frequency, bandwidth filtering 0.05–100 Hz) by marking the earliest onset of the P‐wave in any lead of the frontal plane, and the lead where the end of P‐wave is recorded (especially lead I, V5 and V6, and aVR and in some cases V1 [sagittal force]) (see *Figure* *3*). The interval between these two points (it is recommended to trace vertical lines) will be the total P‐wave duration (*Figure* *3A*, Steps 2a and 3a). While the onset of the atrial electrical activity is usually clearly seen, the end of atrial electrical activity is more difficult. It can be complicated by small amplitude electrical activity, in particular in atria with inhomogeneous electrical propagation and different conduction properties such as seen in patients with atrial cardiomyopathy (AtCM). These small atrial electrical activities can be filtered out by routine filtering techniques. It should be noted that measurement of the P‐wave can underestimate the real duration, especially if the P‐wave has low amplitude.

Several more sophisticated techniques are available to measure the P‐wave duration by means of ECG amplification or by calculating a signal‐averaged P‐wave. When performing an amplified P‐wave duration (aPWD) measurement, it is paramount to amplify the digital ECG recording to visualize also the low amplitude parts of the P‐wave in patients with AtCM. To achieve a good ECG quality, filter settings should be set to 0.05 to 100 Hz without additional noise filtering at a sample rate of 1000 Hz. For measurement of aPWD, the digital ECG should then be amplified to 40–80 mm/mV with a sweep speed of 100 to 200 mm/s. Calculation of a signal averaged P‐wave requires several P‐waves for a robust result. It is recommended to consider 300 P‐waves which equals approximately 5 min of recording time. The recording should be performed without any applied filter and in high‐resolution (1000 Hz or more). The signal‐averaged P‐wave is calculated as a mean signal out of all recorded unfiltered P‐waves. Any random artefacts superimposed on the unfiltered signal disappear, but small changes in the electrical propagation will be preserved in the resulting signal‐averaged P‐wave (*Figure* *3A*, Steps 2b and 3b).

Inter‐atrial block is defined as a P‐wave of 120 ms or more and is classified into three degrees of block: partial (P‐wave ≥120 ms with normal axis and usually bimodal morphology); intermittent (variable degree of IAB detected on the same ECG recording); and advanced (or third‐degree IAB, with P‐wave ≥120 ms plus biphasic morphology [positive–negative] in the inferior leads II, III and aVF). Some atypical patterns of advanced IAB have been described, by duration (P‐wave <120 ms with biphasic morphology), or by configuration (with ‘positive–negative–positive’ morphology or with the presence of isodiphasic components). Nevertheless, in all cases of advanced IAB there will be a final negative component in aVF, reflecting retrograde activation of the left atrium.

## Appendix 2

**How to perform left atrial echocardiography**

Left atrial (LA) size and function should be measured using a non‐foreshortened view (as much LA length as possible) in the apical four‐chamber and/or two‐chamber view. A good quality electrocardiogram (ECG) trace is mandatory, with clearly visible P‐wave.

**Indexed left atrial volume**

The preferred method to quantify LA size is the volumetric method because it accounts for variations in the shape of the left atrium. There are two different principles with which volumes can be obtained: the area–length method and the method of discs (Simpson method). For both methods, tracing of the atrial cavity is required. LA length is measured from the centre of the connecting line between both sides of the mitral annulus to the LA roof. For Simpson, this is done on a four‐chamber view at end‐systole, shortly before the mitral valve opens. For the biplane approach, which is even more exact, also use a two‐chamber view. The volume obtained should be indexed for body surface area (LAVi).

**Left atrial strain**

We recommend to use a speckle tracking technique and measure atrial function using ECG R‐R gating, with baseline at zero on four‐ or two‐chamber views. Depth should be adjusted to enlarge the left atrium and ensure it is fully visualized, including the roof of the atrium without foreshortening (*Figure* *3B*). The left atrium should be focused in four‐ and/or two‐chamber view, obtained with narrow image sector, in order to improve the definition of the grey‐scale image and frame rate. Use the mid‐wall contour of the atrium cavity from one to another mitral ring point, leaving pulmonary veins and atrial appendix excluded from the contour. Adequate measurements are achieved if the region is around 3 mm thin and covers only the LA myocardium. The zero reference is end‐diastole and multiple or index beats should be analysed where sinus rhythm is not present.
